# Metagenomic insights into short-term legume rotation: modulating potato rhizosphere microbiota to enhance tuber yield and quality

**DOI:** 10.3389/fmicb.2025.1680056

**Published:** 2026-01-14

**Authors:** Mingfu Shi, Aixia Guo, Shuhao Qin, Yichen Kang, Weina Zhang, Xinyu Yang

**Affiliations:** 1 Solid-State Fermentation Resource Utilization Key Laboratory of Sichuan Province, Department of Agriculture Forestry and Food Engineering, Yibin University, Yibin, China; 2 College of Horticulture, Gansu Agricultural University, Lanzhou, China; 3 Gansu Academy of Agricultural Sciences, Lanzhou, China

**Keywords:** potato continuous cropping, legume rotation, metagenomics, soil microbial communities and functions, yield and quality

## Abstract

**Objective:**

This study aims to investigate the effects of legume crop rotation on the rhizosphere microbiota and its potential to improve potato (*Solanum tuberosum* L.) productivity and tuber quality. We specifically focus on the microbial functional potential revealed through metagenomic sequencing under different legume rotation systems in the intensive agricultural region of the Chinese Loess Plateau.

**Methods:**

A five-year field experiment (2018–2022) was conducted to establish three cropping systems: (1) continuous potato monocropping for 5 years (CK), (2) continuous potato cropping for 3 years followed by one-year pea rotation and one-year potato cropping (T1), and (3) continuous potato cropping for 3 years followed by one-year faba bean rotation and one-year potato cropping (T2). The impacts of these rotation regimes on potato yield formation, tuber quality, and rhizosphere microbial communities were systematically evaluated, with a focus on microbial diversity and functional potential, using metagenomic sequencing and network analysis.

**Results:**

Metagenomic analysis demonstrated that legume rotation, particularly the T2 system, significantly enriched the relative abundances of Actinobacteria (38.31%) and Proteobacteria (28.40%) in the potato rhizosphere while reducing Acidobacteria (10.03%). Functional annotation further revealed that T2 promoted the expression of microbial genes involved in carbon fixation (K00626, K01895, etc.), nitrogen assimilation (narB, narA, etc.), and sulfur metabolism (cysNC, cysN, etc.), enhanced potential for nutrient cycling. Co-occurrence networks revealed Actinobacteria and Acidobacteria as keystone taxa forming robust interaction modules potentially linked to soil ecological stability. Compared to CK, T2 increased the commercial tuber rate by 85.82%, overall tuber yield by 28.38%, starch content by 34.85%, and vitamin C content by 30.79%, while reducing sugar levels decreased by 9.35%.

**Conclusion:**

Faba bean-potato rotation (T2) effectively mitigated the adverse impacts caused by continuous potato cropping by altering the rhizosphere microbial structure and enhancing microbial functional pathways related to nutrient cycling. This study provides a detailed metagenomic perspective on the microbial mechanisms underlying the benefits of crop rotation and offers a theoretical basis for developing microbiome-informed ecological management strategies to mitigate continuous cropping obstacles in potato production on the Loess Plateau.

## 1 Introduction

Soil microorganisms are fundamental drivers of nutrient cycling, soil fertility maintenance, and plant health, forming complex interactions within the rhizosphere that directly influence agricultural productivity (Bacmaga et al., 2018; Hemkemeyer et al., 2021). Soil microorganisms are integral to material cycling and energy transfer in soils, playing a pivotal role in regulating ecosystem functionality and biogeochemical processes (Delmont et al., 2011). Compared to other habitats, soil microbial communities exhibit greater species richness and more complex community structures. As the most dynamic elements of soil, these microorganisms fulfill essential functions in maintaining soil processes and ensuring ecosystem resilience (Qin et al., 2017a,b; Wang P. et al., 2022; Wang X. et al., 2022). In natural environments, soil microorganisms drive nutrient cycling and transformations, while also preserving soil fertility, structure, and plant health (Bennett et al., 2012; Tiemann et al., 2015; Zheng et al., 2019; Zuo et al., 2023).

Soil microorganisms primarily comprise bacteria, archaea, and fungi, collectively accounting for over 80% of all soil microorganisms (Fadiji et al., 2021; Souza et al., 2013). Bacteria and archaea participate in nutrient mineralization, soil fertility regulation, and environmental remediation, as well as promoting plant growth and suppressing pathogens. Therefore, they are widely recognized as important indicators in soil ecological studies (Reji et al., 2022). Archaea demonstrate resilience to environmental stressors and are actively involved in methane metabolism, ammonia oxidation, as well as carbon and nitrogen cycling in the soil (Andert and Mumme, 2015; Clark et al., 2021). Fungi play a significant role in the decomposition of soil organic matter and nutrient cycling, engaging in the synthesis and breakdown of organic materials while forming mycorrhizal associations with crops to enhance growth, thereby influencing various biochemical processes within the soil (Li et al., 2019; Mommer et al., 2018).

Extensive research has shown that continuous cropping practices induce substantial alterations in soil microbial activity, diversity, and community structure, potentially impacting soil health and crop productivity (Aparicio and Costa, 2007; Gao et al., 2019). Prolonged continuous cropping leads to reduced fertility and a microbial shift from bacteria to fungi, ultimately diminishing potato yields (Qin et al., 2017a,b). The cultivation of tobacco under continuous cropping conditions significantly reduces the Shannon, Simpson, ACE, and Chao1 indices related to soil bacteria, while also altering community structure (Wang P. et al., 2022; Wang X. et al., 2022). Continuous sweet potato cultivation prompts changes in the soil microbial community, including a marked reduction in the Ascomycota phylum after successive cropping (Gao et al., 2019). Furthermore, beneficial fungal populations, such as *Trichoderma* spp., decline, while harmful fungi like *Verticillium* spp., *Fusarium* spp., and *Anthracnose* spp. increase (Gao et al., 2019). These findings indicate that continuous cropping disrupts soil microbial homeostasis, leading to soil degradation. By contrast, crop rotation is an effective agronomic strategy for improving soil microbial diversity, nutrient status, and ecological stability (Chanyarat et al., 2017; Tiemann et al., 2015). Crop rotation significantly increases relative abundance of bacteria compared to continuous cropping, helping to improve soil health and stability (Wang P. et al., 2022; Wang X. et al., 2022). In a potato-zoysia rotation, zoysia cultivation resulted in an increased abundance of dominant soil bacterial phyla, including Anabaena, Bacillus, Ascomycetes, and Nitrospiraea. Beneficial microbial taxa such as *Sphingomonas* spp., *Salicobacter* spp., *Bacillus* spp., and *Pseudonephridium* spp. also increased, while pathogenic fungi such as *Fusarium*, *Streptosporium*, and *Ledebouriomyces* spp. were significantly reduced (Wang P. et al., 2022; Wang X. et al., 2022). These results indicate that rotational zoysia substantially impacts the microbial community structure in potato continuous cropping fields. Moreover, diverse crop rotation patterns enhance the abundance and diversity of soil bacteria compared to continuous cotton cropping. Notably, the cotton-corn-broccoli rotation significantly increases the population of the soil Ascomycete phylum (Zhao et al., 2023). In crop-legume rotations, the strategic selection of legumes is crucial, as different combinations yield distinct rotational effects (Benitez et al., 2021; Liu et al., 2019). Recent studies underscore the vital role of soil microorganisms in regulating agricultural production and soil physicochemical properties (Bi et al., 2022; Larkin, 2018; Perez-Jaramillo et al., 2019). It is evident that crop succession and rotation significantly influence the structure and diversity of soil microbial communities. However, other factors such as the rhizosphere, plant species, and microbial interactions also play essential roles in shaping soil microbial dynamics. Consequently, further research is warranted to elucidate the mechanisms, processes, and extent of these influences on soil microbial communities. Thus, investigating soil microorganisms offers substantial potential for advancing the understanding of the impacts of microbial diversity on plant growth and soil ecosystem functioning.

Metagenomics has emerged as a powerful tool for exploring the complexities of soil microbial communities. Unlike traditional culturing methods, metagenomic sequencing allows for the comprehensive analysis of microbial populations, providing insights into both cultivable and uncultivable microorganisms (Valencia et al., 2018; Wani et al., 2022; Zhao et al., 2025). This approach utilizes high-throughput sequencing to analyze the entire genetic material within a sample, offering a detailed view of microbial diversity, functional gene profiles, and metabolic pathways in various environments, including agricultural soils. Metagenomic technologies have revolutionized our understanding of microbial dynamics in soil ecosystems, enabling the identification of novel microbial species, functional genes, and their contributions to soil processes (Gao et al., 2021; Wani et al., 2024). These advancements have substantial applications in agricultural sciences, particularly in soil health management, pest control, and nutrient cycling, as well as providing new avenues for sustainable farming practices (Perez-Jaramillo et al., 2019; Wani et al., 2022; Zhao et al., 2025). By leveraging metagenomic techniques, researchers can gain deeper insights into microbial interactions within the rhizosphere, helping to optimize crop rotation strategies for enhancing soil fertility and crop productivity (Bai et al., 2025; Hagh-Doust et al., 2023).

This study utilized metagenomics to evaluate the composition, structure, and function of microbial communities in these rotation soils. Furthermore, the investigation focused on the principal metabolic pathways regulated by microbes to thoroughly evaluate how different legume-potato rotations affect the structure of soil microorganisms and their functional genes. The objective of this research is to provide microbiological insights that enhance soil quality by elucidating the potential molecular mechanisms underlying legume rotations, thereby addressing challenges related to continuous cropping disorders in potatoes.

## 2 Materials and methods

### 2.1 Overview of the experimental region and design

**Figure 1:**
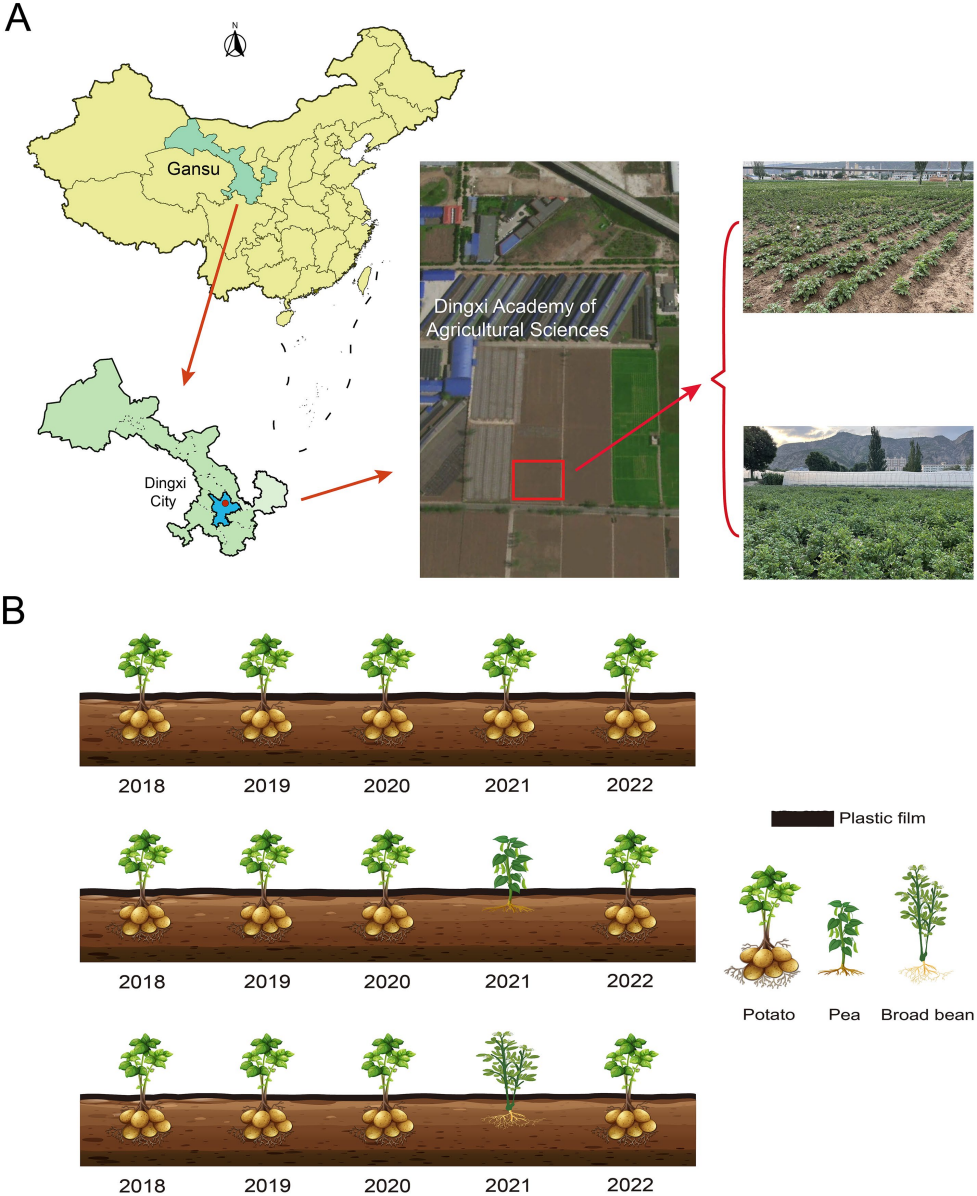
The schematic illustration of the experimental region and planting pattern diagram. **(A)** Denotes the geographical location and aerial view map of the test site; **(B)** showcases the planting layout from 2018 to 2022.

The experiment was conducted at the Dingxi Experimental Station of Gansu Agricultural University in Dingxi City, Gansu Province (104.35′E, 35.33’N), located at an altitude of 1920 m. This semi-arid, rain-fed agricultural area experiences an average annual temperature of 6.4 °C and annual precipitation of 415.2 mm, with more than 56% of precipitation occurring in the autumn months. The soil is classified as loess soil, which is well-suited for potato cultivation (Shi et al., 2023). The field experiment, carried out from 2018 to 2022, investigated three cropping systems: continuous potato monocropping (CK), potato-pea rotation (T1), and potato-faba bean rotation (T2). A randomized block design with three replicates for each treatment was employed (Figure 1). Each plot measured 4.5 m × 8 m, and potato plants were sown at a density of 4,330 plants per 667 m^2^, with row and plant spacing of 40 cm and 28 cm, respectively. Fertilization rates prior to sowing were 300 kg·ha^−1^ of urea (46% N), 250 kg·ha^−1^ of calcium superphosphate (16% P_2_O_5_), and 200 kg·ha^−1^ of potassium sulfate (K_2_O). No irrigation or additional fertilizers were applied during the growth period.

### 2.2 Soil sampling and collection

Soil samples were collected on August 20, 2022, during the tuber expansion phase of potato growth. Five randomly selected potato plants from each plot were excavated using a sanitized shovel. The roots were then gently shaken to collect the surrounding soil, which was transferred into a sterile bag. The samples were sieved through a 1 mm mesh to remove impurities, then transferred into sterile tubes, flash-frozen in liquid nitrogen, and stored at −80 °C until DNA extraction for sequencing analysis. To avoid cross-contamination, tools were sterilized between treatments (Sreejata et al., 2018; Valencia et al., 2018). Details on the nutrient content of the topsoil (0–20 cm) are provided in Supplementary Table S1.

### 2.3 DNA extraction and sequencing

Soil DNA was extracted using the MP FastDNA^®^ SPIN Kit (United States), followed by quantification of concentration with a spectrophotometer (NanoDrop ND2000, United States) and assessment of purity via 1% agarose gel electrophoresis (Biowest agArose, Spain). The agarose gel electrophoresis image of the extracted metagenomic DNA can be found in the Supplementary data. DNA was then fragmented using the Covaris M220 Ultrasonicator to achieve an approximate fragment size of 350 bp. These fragments were used for library construction with the NEXTFLEX Rapid DNA-Seq Kit (Bioo Scientific, United States). Sequencing was performed on the Illumina NovaSeq X Plus platform, ensuring that the sequencing process followed the platform’s standard protocols. Reads shorter than 50 bp or with an average base quality value below 20 were filtered out, along with those containing N bases, using fastp software.^1^ High-quality paired-end and single-end reads were retained and quality-filtered. The filtered reads were then assembled using MEGAHIT v1.1.2 (minimum contig length ≥ 300 bp; https://github.com/voutcn/megahit). Gene prediction was performed using Prodigal v2.6.3, and the predicted gene sequences were clustered with CD-HIT v4.6.1^2^, selecting the longest gene in each cluster as the representative sequence to construct a non-redundant gene set. Gene abundance in each sample was quantified using SOAPaligner (soap2.21release, http://soap.genomics.org.cn/).

### 2.4 Taxonomic and functional annotation

The amino acid sequences of the non-redundant gene set were compared against the NR database^3^ and KEGG database^4^ using Diamond^5^ for species annotation in NR. The abundance of each species was calculated by summing the abundances of its corresponding genes. The COG (Clusters of Orthologous Groups) functions associated with these genes were also determined, and the abundance was calculated similarly by summing the corresponding gene abundances. Additionally, KEGG functions corresponding to each gene were obtained, with the abundance of functional categories determined by summing the abundances of genes associated with KO, Pathway, EC, and Module. Based on the results of metagenomic KEGG annotation, genes involved in carbon, nitrogen, and sulfur metabolism were identified. Specifically, genes encoding ko01200 (Carbon fixation), ko00910 (Nitrogen metabolism), and ko00920 (Sulfur metabolism) at the KO level were selected to establish a gene set for subsequent analysis.

### 2.5 Potato tuber yield and quality determination

For the potato tuber yield and quality analysis, 10 plants were randomly selected from each test group after harvest. The selection criteria for the plants were based on uniform growth stages and visible health, with similar root development. The quantity of tubers per plant was evaluated, and the yield composition was categorized based on tuber size: large potatoes (above 250 g), medium potatoes (50–250 g), and small potatoes (below 50 g). The overall yield was calculated from individual plot harvests. The content of starch, reducing sugars, vitamin C (Vc), and soluble protein in the tubers was determined using a near-infrared quality analyzer (FOSS, NIRS DS 2500, Denmark) (Zhang J. X. et al., 2022; Zhang R. Y. et al., 2022).

### 2.6 Statistical and bioinformatics analysis

The data were analyzed on the online platform of Majorbio Cloud Platform^6^ (Ren et al., 2022). The NR species annotation and KEGG annotation information were analyzed using Circos graphing, principal coordinate analysis (PCoA), group comparison tests, and species and functional contribution analyses. Network analysis illustrating the distribution of samples and species was generated using Cytoscape software.^7^ Cluster analysis was employed to identify key species and pathways associated with microbial community composition and function across the different samples. Statistical analysis of potato yield and quality data was performed using one-way analysis of variance (ANOVA), followed by Tukey’s post-hoc test to identify significant differences among treatment groups. The data were expressed as means ± standard error (SE), and a significance level of *p* < 0.05 was considered significant. Graphic refinement and pathway visualization were accomplished using Adobe Illustrator CC 2018 (Adobe Inc., San Jose, CA, United States).

## 3 Results

### 3.1 Metagenomic sequencing data and microbial community structure analysis

After Illumina sequencing, a total of 667,806,536 raw reads were obtained from nine sample libraries. Following quality filtering, 655,469,922 clean reads were identified, with high-quality sequences in each library exceeding 98.15%. The coverage for all samples consistently surpassed 98%, confirming the accuracy of the microbial community composition reflected in the sequencing data (Supplementary Table S2). A total of 6,774,063 contigs were identified, with a combined length of 3,408,774,120 bases. The N50 statistics showed that over half of the contigs exceeded 497 bp in length, with the longest cluster measuring 109,644 bp and the shortest at 300 bp (Supplementary Table S2). Non-redundant gene catalogs were constructed for bacteria (3,679,802 genes), archaea (70,271 genes), and fungi (1,987 genes).

The metagenomic sequencing results were aligned with the NR database, and the microbial community was analyzed at the domain level (Supplementary Figure S1). The results indicated that bacteria dominated the microbial community (94.95%), followed by archaea (4.81%), eukaryotes (0.15%), viruses (0.03%), and others. The highest proportion of bacteria was observed in all treatments, with CK, T1, and T2 showing values of 94.08, 95.12, and 96.23%, respectively (Supplementary Figure S1). Overall, the study identified 4 domains, 9 kingdoms, 197 phyla, 341 orders, 620 families, 1,234 genera, and 27,163 microbial species at various taxonomic levels.

### 3.2 Analysis of soil microbial community composition at phylum and genus levels

**Figure 2:**
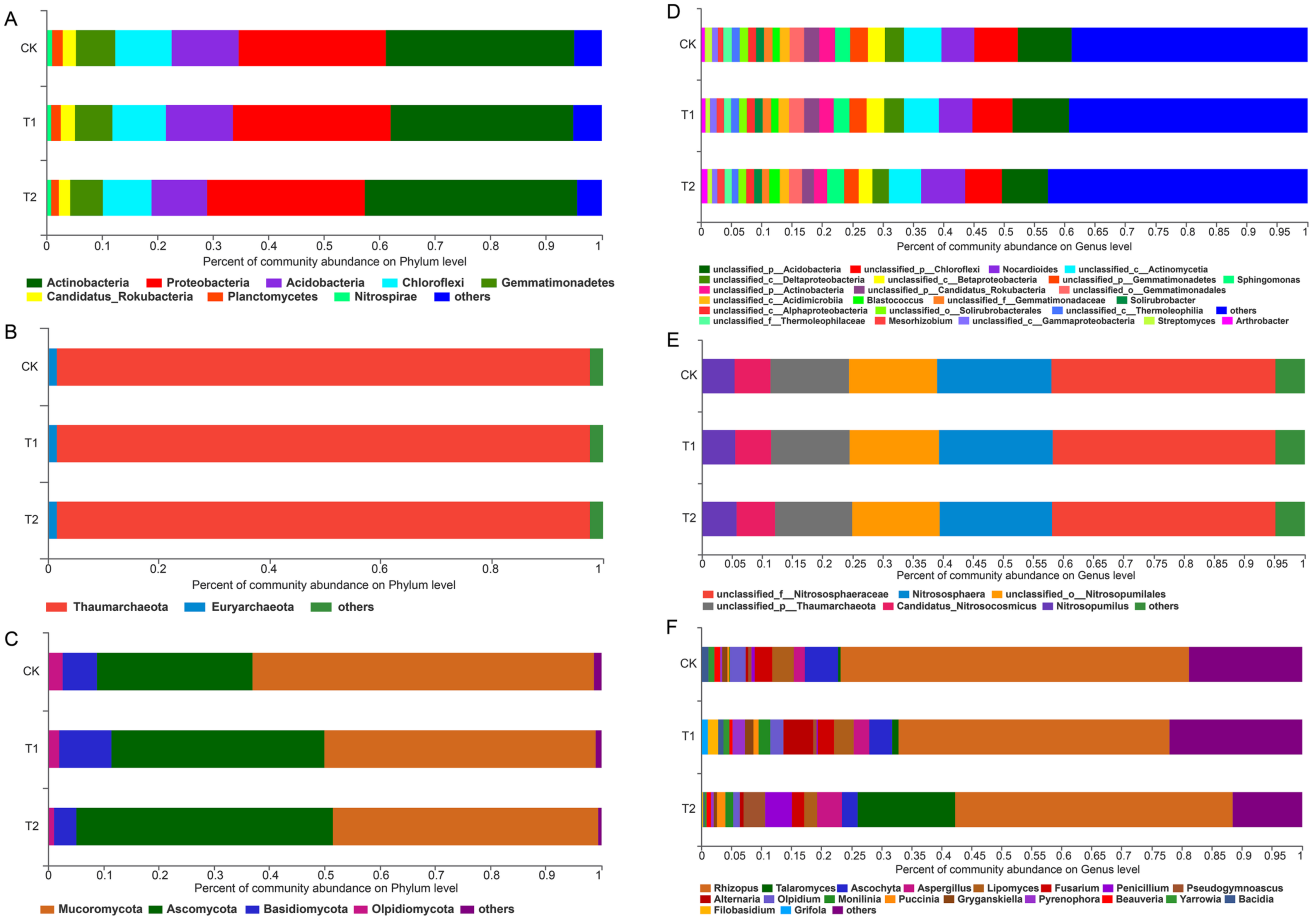
Soil microbial community composition (phylum and genus level). **(A–C)** Denote the phylum levels of bacteria, archaea, and fungi, respectively. **(D–F)** Denote the genus levels of bacteria, archaea, and fungi, respectively.

At the phylum level, the microbial communities across all treatments exhibited a similar composition (Figure 2). The dominant bacterial phyla included *Actinobacteria*, *Proteobacteria*, *Acidobacteria*, *Chloroflexi*, and *Gemmatimonadetes*, with *Actinobacteria* being the most abundant in all treatments. The relative abundance of *Actinobacteria* was highest in T2 (38.31%), while *Proteobacteria* increased gradually in T1 and T2, peaking at 28.40% in T2. *Acidobacteria* showed a decreasing trend in T2, with a relative abundance of 10.03%. *Chloroflexi* and *Gemmatimonadetes* exhibited a consistent decline from CK to T1 and T2 (Figure 2A).

Among archaea, *Thaumarchaeota* was the most abundant phylum, representing 96.19–96.22% across treatments, with a slight increase in T1 and T2. The relative abundance of *Euryarchaeota* was higher in T2, increasing by 1.85 and 4.43% compared to CK and T1, respectively (Figure 2B).

In the fungal community, *Mucoromycota* and *Ascomycota* were the dominant phyla. The relative abundance of *Mucoromycota* decreased in T1 and T2 by 20.57 and 22.27%, respectively, while *Ascomycota* increased by 36.87 and 64.77%. These changes indicate that legume crop rotation alters fungal community composition, which may affect soil microbial dynamics (Figure 2C).

The microbial communities at the genus level were analyzed for each treatment (Figure 2D). The top 10 bacterial genera across all treatments included *unclassified_p_Acidobacteria*, *unclassified_p_Chloroflexi*, *Nocardioides*, *unclassified_c_Actinomycetia*, *unclassified_c_Deltaproteobacteria*, *unclassified_c_Betaproteobacteria*, *unclassified_p_Gemmatimonadetes*, *Sphingomonas*, *unclassified_p_Actinomycetia*, and *unclassified_p_Candidatus_Rokubacteria*.

In the T1 treatment, the relative abundance of *unclassified_p_Acidobacteria* was lowest (7.60%), and both T1 and T2 treatments showed a decrease in *unclassified_p_Chloroflexi* by 6.50 and 6.61%, respectively. Similarly, *unclassified_c_Actinomycetia* decreased by 14.12% in T1 and 15.00% in T2. In contrast, *Nocardioides* and *Sphingomonas* increased by 1.83 and 34.43% in T1, and 4.84 and 14.52% in T2, respectively. Additionally, *unclassified_c_Deltaproteobacteria*, *unclassified_c_Betaproteobacteria*, *unclassified_p_Gemmatimonadetes*, *unclassified_p_Actinomycetia*, and *unclassified_p_Candidatus_Rokubacteria* showed decreases of 14.42, 15.71, 19.05, 16.60, and 21.91% in T2 compared to CK.

For archaea, dominant genera included *unclassified_f_Nitrososphaeraceae*, *Nitrososphaera*, *unclassified_o_Nitrosospumilales*, *unclassified_p_Thaumarchaeota*, *Candidatus_Nitrosocosmicus*, and *Nitrosopumilus* (Figure 2E). The relative abundance of *unclassified_f_Nitrososphaeraceae* and *Nitrososphaera* was higher in CK compared to T1 and T2. In T1, *unclassified_o_Nitrosospumilales* and *unclassified_p_Thaumarchaeota* were more abundant than in CK and T2, while *Candidatus_Nitrosocosmicus* and *Nitrosopumilus* showed higher relative abundances in T2.

Fungal genera were dominated by *Rhizophagus*, *Talaromyces*, *Ascochyta*, *Aspergillus*, *Lipomyces*, *Fusarium*, *Penicillium*, *Pseudogymnoascus*, *Alternaria*, *Olpidium*, and *Monilinia* (Figure 2F). In both T1 and T2 treatments, the relative abundance of *Rhizophagus*, *Ascochyta*, *Lipomyces*, *Fusarium*, and *Olpidium* decreased compared to CK, while *Talaromyces*, *Aspergillus*, *Pseudogymnoascus*, and *Monilinia* within the *Penicillium* genus increased. Notably, *Alternaria* was more abundant in T2 compared to both CK and T1.

### 3.3 Hierarchical clustering and principal coordinates analysis of soil microorganisms

**Figure 3:**
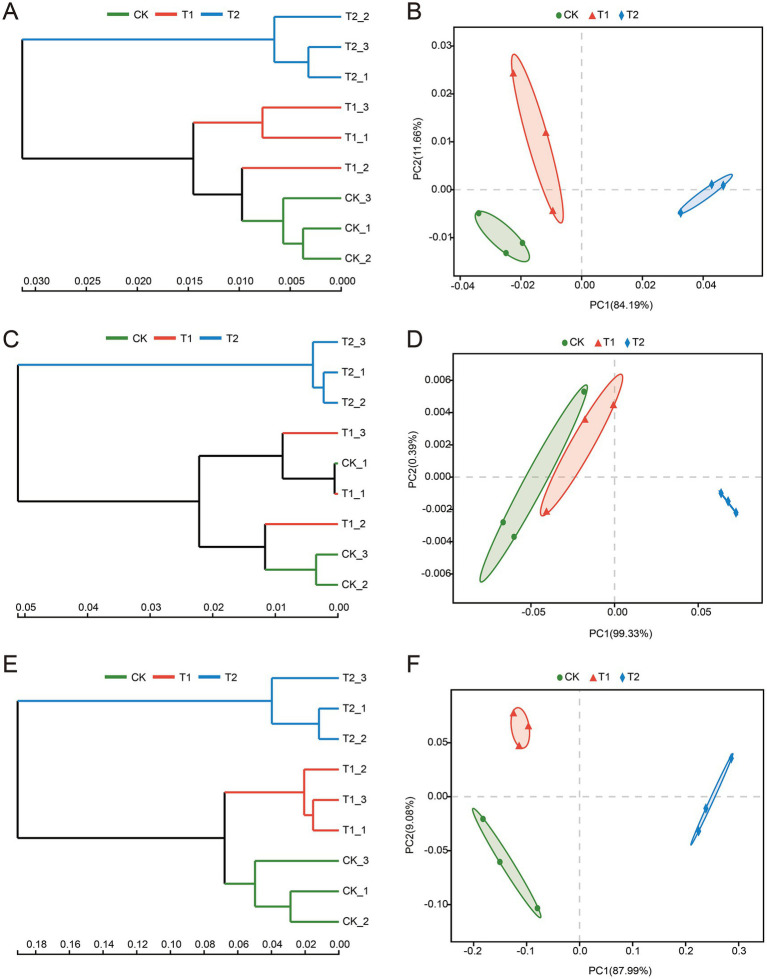
Hierarchical clustering and principal coordinate analysis (PCoA) of soil microbial communities at the phylum level. **(A,C,E)** Represent the dendrograms of bacteria, archaea, and fungi at the phylum level, respectively, with branch lengths indicating the dissimilarity between samples. **(B,D,F)** Represent the PCoA plots of bacteria, archaea, and fungi at the phylum level.

Beta diversity was assessed using Bray-Curtis distance matrices, followed by hierarchical clustering with the Unweighted Pair Group Method with Arithmetic Mean (UPGMA) (Figure 3). The results revealed greater dissimilarity in microbial communities (bacteria, archaea, and fungi) in the T2 treatment compared to CK and T1, with T2 samples showing clearer separation. In contrast, microbial communities in CK and T1 were more similar (Figures 3A,C,E).

Principal Coordinates Analysis (PCoA) of bacterial communities explained 95.85% of the variation, with PC1 and PC2 accounting for 84.19 and 11.66%, respectively. The samples clustered into three distinct groups, with CK and T1 samples showing closer proximity, indicating similar bacterial community structures (Figure 3B).

PCoA of archaeal communities revealed that T1 samples were more similar to CK, while T2 samples exhibited significant divergence, suggesting that faba bean rotation notably influenced archaeal community composition (Figure 3D). For fungal communities, PCoA analysis explained 97.07% of the variance, with PC1 and PC2 contributing 87.99 and 9.08%, respectively, further indicating distinct microbial compositions between CK, T1, and T2 treatments (Figure 3F).

### 3.4 Analysis of differential microorganisms and network analysis among microbial communities

**Figure 4:**
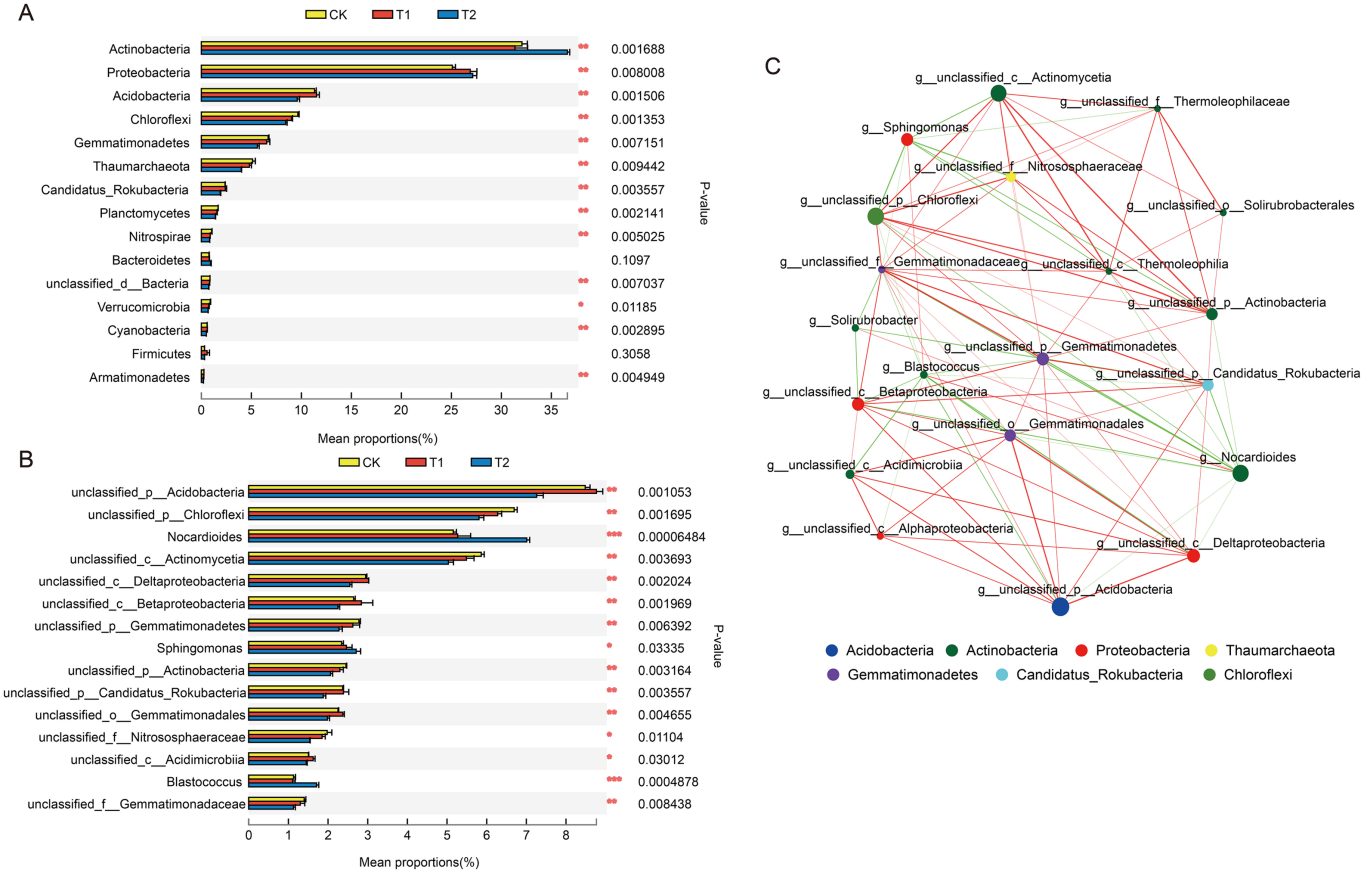
Differential analysis of soil microbial communities at the phylum and genus levels and correlation network analysis at the genus level. **(A)** Represents the analysis of species differences among the top 15 microbial groups in each treatment at the phylum level; **(B)** is the analysis of species differences among the top 15 microbial groups ranked in each treatment at the genus level; **(C)** is a network diagram for microbial genus level correlation analysis, where the size of nodes in the graph represents the abundance of species, and the larger the abundance value, the larger the nodes. The red line represents a positive correlation between species, while the green line represents a negative correlation between species; The thickness of the line indicates the magnitude of the correlation coefficient value.

Species differences were analyzed for the top 15 microbial taxa ranked at the phylum level across treatments (Figure 4A). The results indicated significant differences (*p* < 0.05) in the abundances of Actinobacteria, Proteobacteria, Acidobacteria, Chloroflexi, Gemmatimonadetes, Thaumarchaeota, Candidatus_Rokubacteria, Planctomycetes, Nitrospirae, unclassified_d_Bacteria, Cyanobacteria, and Armatimonadetes among the treatments, with Verrucomicrobia also exhibiting significant differences.

At the genus level, the analysis of the top 15 dominant genera revealed significant variations among *unclassified_p_Acidobacteria*, *unclassified_p_Chloroflexi*, *unclassified_c_Actinomycetia*, *unclassified_c_Deltaproteobacteria*, *unclassified_c_Betaproteobacteria*, *unclassified_p_Gemmatimonadetes*, unclassified_p_Actinomycetia, *unclassified_p_Candidatus_Rokubacteria*, *unclassified_o_Gemmatimonadetes*, and *unclassified_f_Gemmatimonadaceae*, all showing high significance (*p* < 0.01) (Figure 4B). *Nocardioides* and *Blastococcus* also demonstrated significant differences (*p* < 0.05), while *Sphingomonas*, *unclassified_f_Nitrososphaeraceae*, and *unclassified_c_Acidimicrobiia* displayed noteworthy variations.

The top 20 species, based on total abundance at the genus level, were selected for correlation analysis and network mapping (Figure 4C). The network showed significant interactions among genera, with a transitivity of 0.7122, a network diameter of 4, an average shortest path length of 1.63, and a total of 95 edges, including 64 positive and 31 negative correlations. This indicates strong microbial interactions across the three treatments. Analysis of node degree centrality revealed five core genera: *Gemmatimonadaceae*, *Gemmatimonadetes*, *Nocardioides*, *Candidatus Rokubacteria*, and *Blastococcus*, which exhibited the highest centrality values. In contrast, genera such as *Alphaproteobacteria*, *Solirubrobacter*, and *Solirubrobacterales* showed weaker correlations. The most interconnected phyla, *Actinobacteria*, *Gemmatimonadetes*, and *Acidobacteria*, accounted for 50.53, 17.89, and 14.74% of the linkages, respectively, highlighting their central roles within the microbial community.

### 3.5 KEGG functional annotation and microbial function analysis

**Figure 5:**
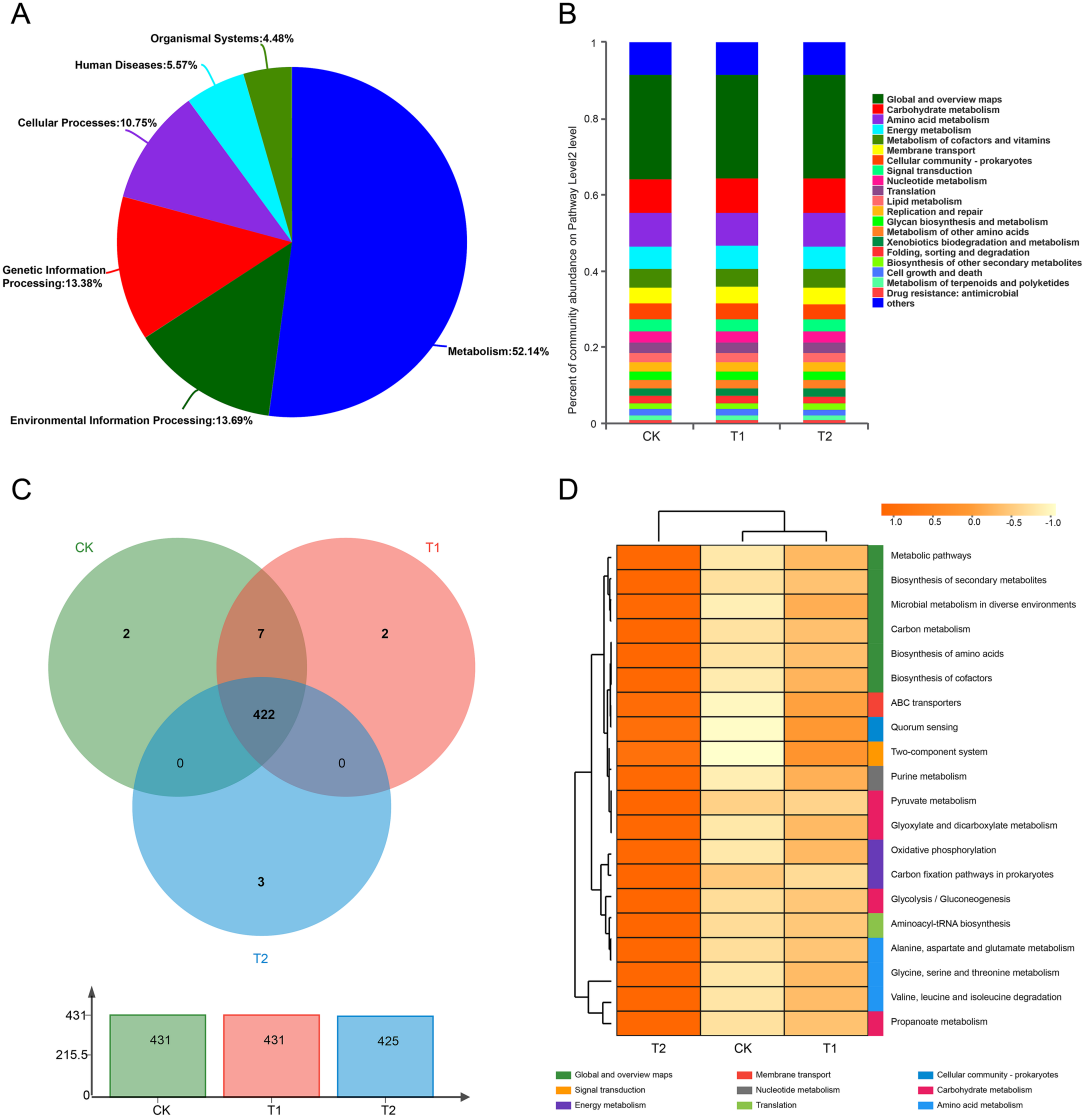
KEGG functional annotation and analysis of soil microorganisms. **(A)** Represents the primary pathway for KEGG functional annotation; **(B)** is the KEGG functional annotation secondary pathway; **(C,D)** are KEGG functional annotation tertiary pathways.

Soil microbial functional genes were annotated using the KEGG database, revealing distinct functional profiles across treatments. The primary KEGG pathways included Metabolism (52.14%), Environmental Information Processing (13.69%), and Genetic Information Processing (13.38%), with additional categories such as Cellular Processes (10.75%) and Human Diseases (5.57%) also notable (Figure 5A). At the secondary pathway level (Figures 5B,C), metabolic pathways such as Carbohydrate Metabolism, Amino Acid Metabolism, and Energy Metabolism were prevalent across treatments, with 431 class 3 pathways annotated, showing a similar number across treatments (431 for CK, 431 for T1, and 425 for T2). A total of 422 unique pathways were identified, with 2, 2, and 3 pathways specific to CK, T1, and T2, respectively. Notably, T2 exhibited distinct differences in KEGG tertiary annotations, with the Biosynthesis of Secondary Metabolites, Microbial Metabolism in Diverse Environments, Carbon Metabolism, and Biosynthesis of Amino Acids identified as dominant pathways (Figure 5D).

Furthermore, functional analysis using the COG and KEGG databases identified 24 COG functional types and 53 KEGG pathways, with the top 15 functional categories highlighted based on relative abundance (Supplementary Figure S2). Enriched COG categories included Amino Acid Transport and Metabolism, Carbohydrate Transport and Metabolism, Energy Production and Conversion, and Signal Transduction Mechanisms. The most abundant KEGG pathways comprised Metabolic Pathways, Biosynthesis of Secondary Metabolites, and Microbial Metabolism in Various Environments, while pathways like Carbon Metabolism, Amino Acid Biosynthesis, and Quorum Sensing exhibited significant differences between treatments. These findings indicate a consistent microbial functional metabolism across treatments, with distinct differences observed in the biosynthesis and metabolism of metabolic pathways, amino acid biosynthesis, and quorum sensing.

### 3.6 Analysis of carbon, nitrogen, and sulfur cycling pathways and related enzyme gene abundance

**Figure 6:**
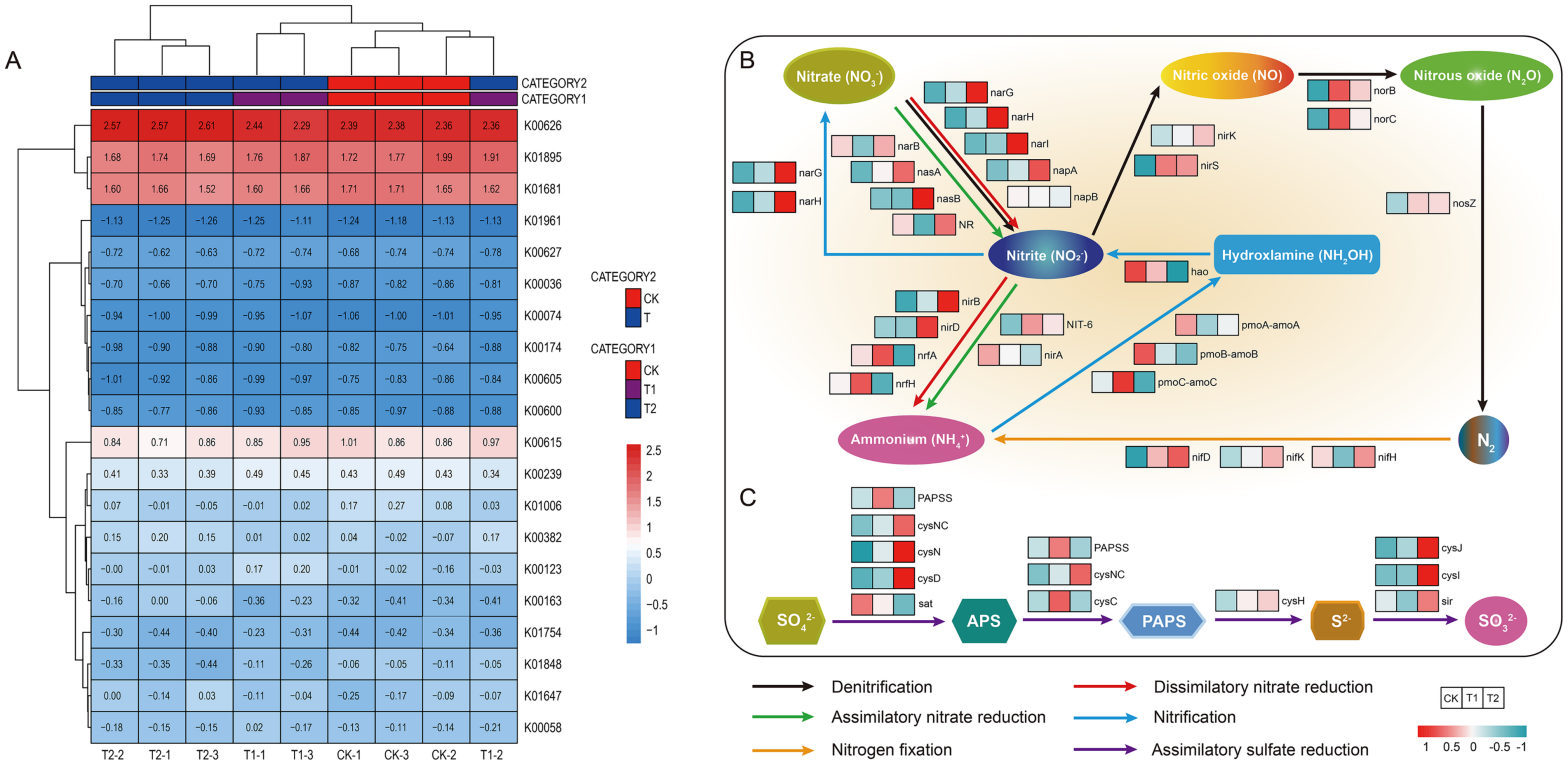
Differences in gene sequence abundance of microbial carbon, nitrogen, and sulfur metabolism pathways. **(A)** Represents the difference in carbon metabolism gene sequence abundance; **(B)** represents the nitrogen metabolism-related pathways; **(C)** represents the sulfur metabolism-related pathways.

Key metabolic pathways, including carbon, nitrogen, and sulfur metabolism, were analyzed and annotated in the soils of the three treatments (Supplementary Table S2). The top 20 carbon metabolism-related functional genes, ranked by abundance across treatments, were analyzed using a heatmap (Figure 6A). The composition of these genes was consistent across all treatments, though their abundance varied. The primary functional genes included K00626, K01895, K01681, and K00615. Specifically, the K00626 gene was primarily involved in the ethylmalonyl pathway (M00373), hydroxybutyrate dicarboxylate cycle (M00374), and hydroxypropionic acid hydroxybutyrate cycle (M00375). K01681 was associated with the citrate cycle (M00010), reduced citrate cycle (M00173), and citric acid cycle (M00009), while K01895 was mainly linked to methane synthesis (M00357). In the nitrogen metabolism pathway (Figure 6B), most genes related to assimilatory nitrate reduction, except for *narA*, showed higher abundance in T2, with *narB*, *nasA*, *nasB*, *NR*, *NIT-6*, and *nirA* being prominent. For dissimilatory nitrate reduction, genes such as *narG*, *narH*, *narI*, *napA*, *nirB*, and *nirD* exhibited decreased abundance in CK but increased in T2. Denitrification genes (*nirK*, *nirS*, *norBC*, and *nosZ*) generally showed increased abundance in T1 and T2. The *nirK* and *nirS* genes encode different nitrite reductases, with distinct roles in the denitrification process. In nitrogen fixation, *nifD*, nifK, and nifH were more abundant in T2, whereas nitrification genes (*pmoB*, *pmoA*, and *hao*) were more prevalent in CK compared to T1 and T2. In sulfur metabolism (Figure 6C), genes related to assimilatory sulfate reduction (*cysNC*, *cysN*, *cysD*, *cysJ*, *cysI*, and *sir*) increased in T2, while sat decreased. *PAPSS* and *cysC* were elevated in T1.

### 3.7 Effects of legume crop rotation on potato yield and tuber quality

**Figure 7:**
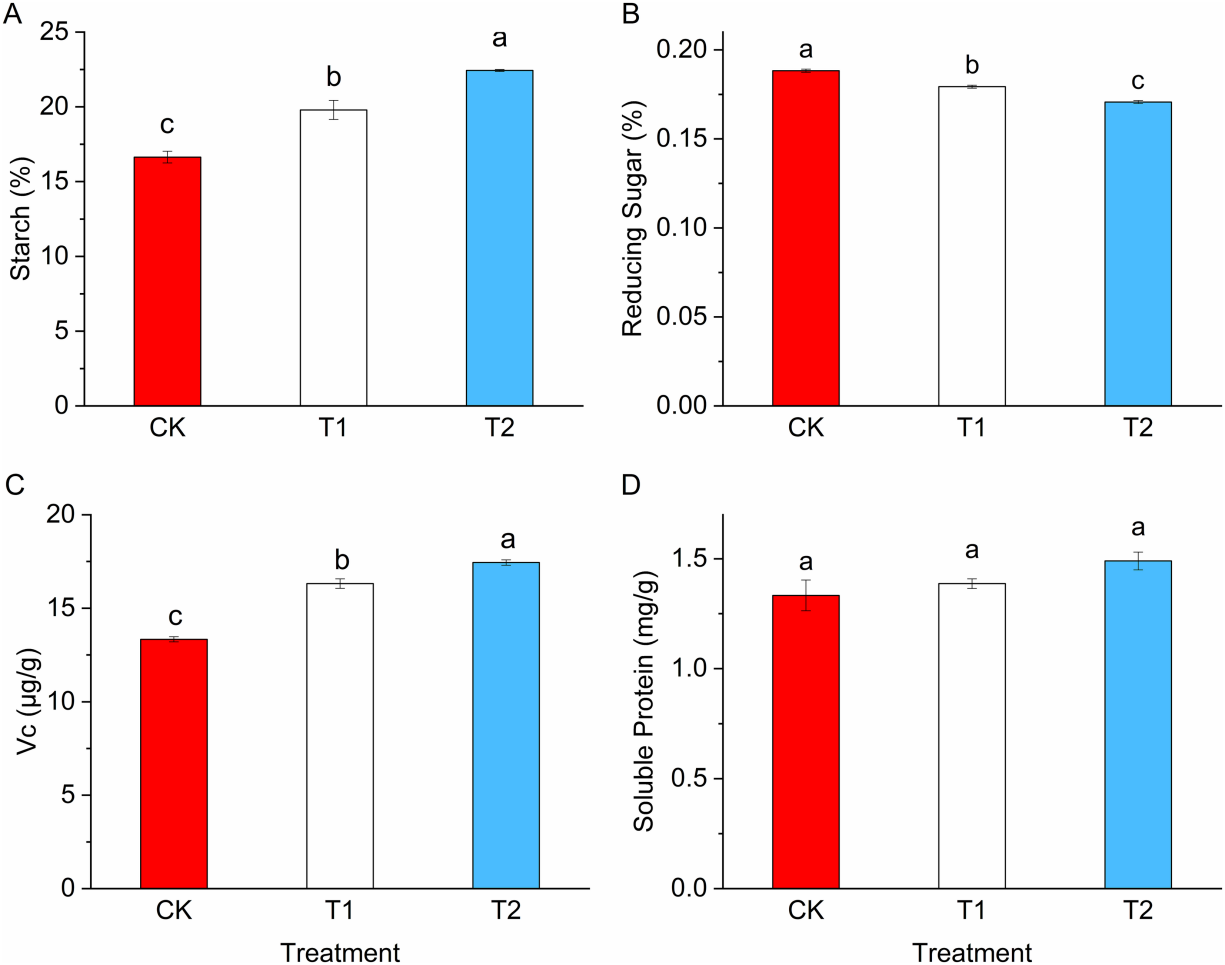
Effect of legume rotation on potato tuber quality. **(A)** Represents starch; **(B)** represents reducing sugar; **(C)** represents Vc; **(D)** represents soluble protein.

According to Table 1, the proportion of large potatoes and overall potato yield exhibited significant increases following the rotation with leguminous crops. Specifically, the ratio of large potatoes in treatments T1 and T2 rose by 59.06 and 85.82%, respectively, while yields increased by 25.13 and 28.38%. No significant differences were noted in the ratios of medium, small, or commercial potatoes among the various treatments. Crop rotation markedly affects the quality of potato tubers (Figure 7). Compared to the control group (CK), the starch and vitamin C contents increased substantially by 18.98 and 22.37% in T1, and by 34.85 and 30.79% in T2, respectively (Figures 7A,C). Reducing sugar levels were significantly lower than those in CK, with reductions of 4.78 and 9.35%, respectively (Figure 7B). Soluble protein content showed no significant variation across the treatments.

**Table 1:** Effects of legume rotation on potato yield composition.

Treatment	The ratio of large potatoes (%)	The ratio of medium potatoes (%)	The ratio of small potatoes (%)	The ratio of commercial potatoes (%)	Yield (kg·ha^−1^)
CK	20.13 ± 2.03b	25.98 ± 5.83a	21.58 ± 2.44a	0.68 ± 0.04a	16930.91 ± 812.49b
T1	32.01 ± 5.11a	34.39 ± 2.28a	15.63 ± 0.85a	0.81 ± 0.01a	21185.07 ± 571.44a
T2	37.40 ± 1.75a	32.38 ± 6.34a	17.12 ± 3.70a	0.80 ± 0.05a	21735.86 ± 677.56a

### 3.8 Correlation between soil microbial genus-level community composition and potato yield and tuber quality

**Figure 8:**
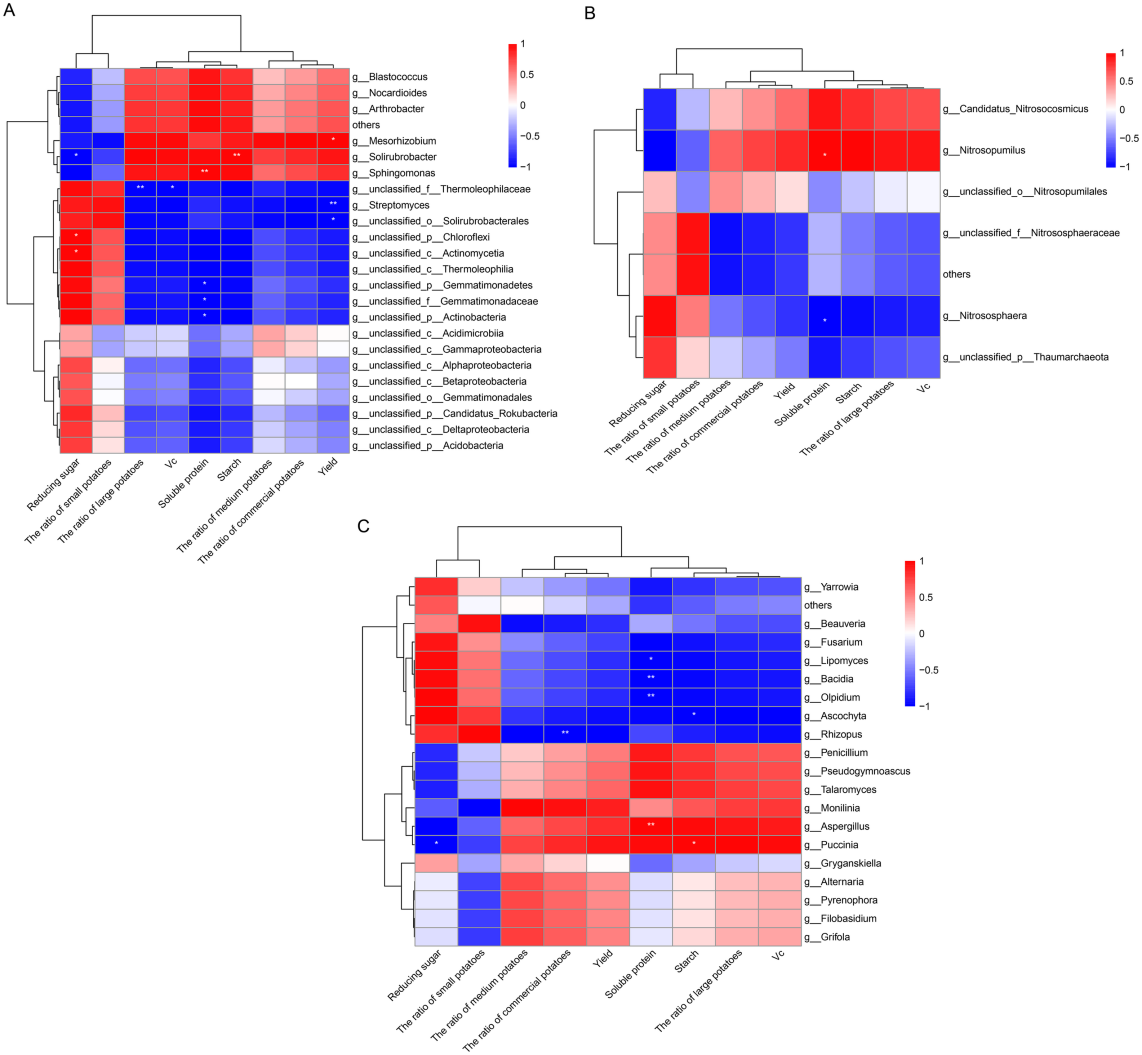
Correlation between soil microbial genes level community composition and potato yield and tuber quality. **(A)** Represents bacteria; **(B)** represents archaea; **(C)** represents fungi.

To explore the relationship between soil microbial communities and potato yield and tuber quality, Pearson correlation analysis was performed on the microbial genus-level community composition and various growth parameters. In the bacterial community (Figure 8A), the abundance of *Nocardioides* and *Sphingomonas* showed positive correlations with potato yield, tuber starch, vitamin C, and soluble protein content. In the archaeal community (Figure 8B), the abundance of *Nitrososphaera* was negatively correlated with potato yield and tuber starch, vitamin C, and soluble protein content, highlighting the important role of archaea in nitrogen cycling and soil fertility improvement. In the fungal community (Figure 8C), the abundance of *Rhizophagus* was strongly correlated with potato tuber quality and yield, showing a significant negative correlation with the proportion of the ratio of commercial potatoes. The abundance of *Aspergillus* was positively correlated with soluble sugar content in potato tubers.

## 4 Discussion

### 4.1 Composition and variation of soil microbial communities

The soil microbial community plays a pivotal role in determining soil quality, acting as a barometer of environmental changes and significantly influencing the ecological diversity within agricultural systems (Bahram et al., 2018; Gong et al., 2019). In this study, we detected a total of 27,163 microorganisms across CK (potato continuous cropping), T1 (pea-potato rotational cropping), and T2 (faba bean-potato rotational cropping) treatments, encompassing 4 domains, 9 kingdoms, 197 phyla, 341 orders, 620 families, and 1,234 genera. Bacteria were the dominant group, constituting over 94% of the total microbial community, which aligns with similar findings from other soil habitats (Bahram et al., 2018; Pang et al., 2021). This dominance of bacteria further highlights their fundamental role in soil ecology, including their impact on plant growth and soil health. Significant shifts in microbial community composition were observed between continuous and rotational cropping systems, particularly at the phylum level, where Actinobacteria and Proteobacteria emerged as predominant groups. These phyla are critical to soil health, with Actinobacteria playing a vital role in organic matter decomposition and pathogen inhibition (Górska et al., 2022; Zhang et al., 2017), while Proteobacteria are key contributors to nitrogen cycling in soils. The influence of legume crop rotations on soil bacterial composition was evident, with enhanced nitrogen uptake facilitated by both Ascomycetes and Actinomycetes (Essel et al., 2019; Jiang et al., 2016). Furthermore, the presence of Thaumarchaeota among archaea corroborated findings from similar studies on maize-planted soils (Megyes et al., 2021), emphasizing the ecological role of archaea in soil nutrient cycling.

The fungal community, dominated by Mucoromycota and Ascomycota, exhibited distinct variations across treatments, with a particularly high abundance of Mucoromycota in CK. While Mucoromycota has been associated with Mucoromycosis, which poses disease risks, its reduced presence in T1 and T2 suggests that pea and faba bean rotations may mitigate these risks (Cruz-Lachica et al., 2018). These findings were supported by a multi-group comparative analysis, which highlighted the differential abundances of microorganisms such as Actinobacteria, Proteobacteria, Acidobacteria, and Thaumarchaeota. These taxa are known to enhance soil health and promote plant growth (Kayikcioglu et al., 2020; Khakbazan et al., 2019; Lupwayi et al., 1999; Wright et al., 2016). The observed differences in microbial phyla between continuous and rotational cropping treatments imply that different cropping systems can increase plant productivity, mitigate diseases, and improve the soil bioenvironment.

At the genus level, dominant bacterial groups included *unclassified_p_Acidobacteria*, *Nocardioides*, *unclassified_c_Actinomycetia*, *unclassified_c_Deltaproteobacteria*, and *Sphingomonas*. These genera are involved in biochemical reactions and metabolic cycles in the soil, benefiting plant growth (Fadiji et al., 2021; Xu et al., 2021; Zhao et al., 2019). Archaeal genera like Nitrososphaera further corroborated the results of prior studies (Clark et al., 2021; Souza et al., 2013). The study also revealed that fungal genera, including Rhizophagus, Talaromyces, and Fusarium, varied in their relative abundance depending on the cropping system, reflecting the inter-root effects of plants on fungal diversity (Chen et al., 2018; Li et al., 2019; Sun et al., 2022). However, the proliferation of pathogens such as *Fusarium* and *Ascochyta*, which can outcompete native species, could alter microbial community dynamics, leading to potential disruptions in soil health (Davidson et al., 2011; Kang et al., 2020). The hierarchical clustering and PCoA analyses demonstrated distinct separation between microbial communities in rotational versus continuous cropping systems, validating significant differences across bacterial, archaeal, and fungal populations.

Correlation network analyses of soil microbial community interactions revealed that genera such as *g__unclassified__f__Gemmatimonadaceae*, *g__unclassified__p__Gemmatimonadetes*, *g__Nocardioides*, *g__unclassified__p__Candidatus_Rokubacteria*, and *g__Blastococcus exhibited* a higher number of network nodes, maximum connections, and aggregation coefficients, indicating a large and complex network. *Nocardioides* and *Sphingomonas* were identified as central hub genera in the co-occurrence network, suggesting that they may play potentially important roles in maintaining microbial community structure. The presence of positive and negative correlations in the co-occurrence network reflects different interactions among microorganisms; positive correlations suggest ecological niche congruence or synergistic effects, while negative correlations may indicate competitive or predatory relationships (Layeghifard et al., 2017). This study reveals that several genera affiliated with Actinobacteria, Gemmatimonadetes, and Acidobacteria exhibit complex co-occurrence patterns, suggesting potential ecological associations within the microbial community. Nevertheless, such network-based relationships do not constitute direct evidence of functional interactions, and therefore the interpretation should be approached with caution.

### 4.2 Soil microbial community functions

Soil microorganisms are vital for nutrient cycling and metabolism (Bora et al., 2022; Gao et al., 2021). This study identified 46 secondary pathways annotated by KEGG, categorized into six primary metabolic pathways: metabolism, environmental information processing, genetic information processing, cellular processes, human diseases, and organic systems. Metabolism, particularly carbohydrate, amino acid, and energy metabolism, contained the highest number of enriched functional genes. These metabolic processes are essential for crop growth and biochemical functions (Pang et al., 2021). Although microbial community structure varied according to planting patterns, microbial functions remained relatively conservative, indicating functional redundancy across treatments, which helps maintain ecosystem stability despite external disturbances (Gao et al., 2021). Pang et al. (2021) found that most functional metabolic pathways among microorganisms in different treatments were similar, though gene sequence variations highlighted the involvement of different microbial species in maintaining stability under varying conditions.

Differential analysis of microbial functions revealed 24 species annotated in the COG database, with significant differences in categories such as functional prediction (R), signal transduction (T), and cell wall biogenesis (M). KEGG annotation revealed significant differences in metabolic pathways, including amino acid biosynthesis, group sensing, and two-component systems. The decomposition of organic matter in soil enhances microbial metabolism, supporting plant growth (Gilbert et al., 2013; Li et al., 2020). The crop rotation treatments (T1, T2) revealed abundant gene sequences for amino acid and carbohydrate metabolism. These treatments favored nitrogen accumulation, evidenced by higher total nitrogen content in the soil, which correlated with increased gene sequences for glutamate synthase and ammonium transport (Souza et al., 2015). Crop rotation also supported energy metabolism, which enhances plant tolerance (Zhang J. X. et al., 2022; Zhang R. Y. et al., 2022). Overall, the functional metabolic profiles across all treatments were consistent, reinforcing the idea that core microbial functions remain conserved, thereby ensuring ecological stability despite external disturbances.

### 4.3 Soil microbial carbon, nitrogen, and sulfur metabolic pathways

Microbial functional genes encode enzymes crucial for nutrient cycling, offering insight into soil nutrient dynamics (Dong et al., 2020; Wani et al., 2025). This study demonstrates that several functional genes related to carbon (C), nitrogen (N), and sulfur (S) metabolism were significantly upregulated in the T2 treatment, thereby enhancing soil nutrient transformations (Hao et al., 2019; Hemkemeyer et al., 2021; Leite et al., 2022). Soil microbes play an essential role in the carbon cycle, influencing carbon fixation, methane metabolism, and degradation (Bardgett et al., 2008; Ma et al., 2023). Specifically, in nitrogen metabolism, genes associated with nitrate reduction, denitrification, and nitrogen fixation were more abundant in the T2 treatment. This underscores the impact of legumes in modulating nitrogen metabolism, which supports organic matter content and helps mitigate the adverse effects of continuous cropping on soil health (Bhattarai et al., 2021; Coskun et al., 2017). Additionally, genes related to sulfur metabolism were elevated in both T1 and T2 treatments, indicating that legume rotations promote the prevalence of sulfur metabolism-related genes. The increased abundance of C, N, and S cycling genes across agricultural soils suggests that agricultural practices disturb the biogeochemical cycling processes, highlighting the need for ecological restoration (Pang et al., 2021; Xiang et al., 2020).

A close correlation exists between microbial community structure and soil microbial functions (Yang et al., 2018). In this study, analysis based on the COG database indicated that the genus *Nocardioides* significantly contributes to key functions, including microbial metabolism, carbon metabolism, and amino acid biosynthesis in various environments. The genus *Nocardioides*, belonging to the Actinobacteria phylum, plays a critical role in inhibiting Fusarium spinosum, potentially through direct antagonism *via* antibiotic production or ecological niche overlap with fungal pathogens (Zhao et al., 2019). Moreover, *Nocardioides* spp. are integral to soil nitrogen cycling, enhanced potential for nutrient cycling processes (Cheng et al., 2021). Further investigation is warranted to understand the composition of root secretions in T1 and T2 treatments and their effects on specific metabolic functions within soil microbial communities.

### 4.4 Effects of legume crop rotation on potato tuber yield and quality

Crop yield and quality are influenced by several factors, including the environment, plant genetics, and agricultural practices (Fang et al., 2021; Wang P. et al., 2022; Wang X. et al., 2022). In the present study, rotations with pea and, in particular, faba bean significantly enhanced potato yield and tuber size, with the faba bean rotation producing the greatest improvement. Consistent with previous studies (Sainju et al., 2020), legume rotations also increased tuber starch and vitamin C contents, indicating a positive impact on both yield and nutritional quality. It is well established that legume crops improve soil fertility through biological nitrogen fixation, thereby increasing nitrogen availability for subsequent crops (Liu et al., 2019; Moulin et al., 2011). Thus, the enhanced potato productivity observed in the faba bean rotation treatment is likely attributable, at least in part, to the direct contribution of biologically fixed nitrogen. In addition, our metagenomic results revealed concomitant shifts in microbial community structure and functional gene profiles under legume rotations. However, these microbial changes may represent a secondary response to altered soil nutrient conditions rather than a direct causal driver of yield improvement.

The correlation analysis revealed key microbial groups influencing potato yield and tuber quality (Figure 8). In the bacterial community, *Nocardioides* and *Sphingomonas* were positively correlated with yield and tuber quality, indicating their role in nutrient cycling and plant growth. These results align with previous studies on their beneficial effects in soil (Fadiji et al., 2021; Xu et al., 2021). *Nitrososphaera*, an archaeal genus involved in nitrogen cycling, showed a negative correlation with yield and quality, suggesting an excess of nitrification may hinder plant growth (Clark et al., 2021; Souza et al., 2013). *Rhizophagus*, a mycorrhizal fungus, was positively correlated with yield and tuber quality, supporting its role in enhancing nutrient uptake (Sun et al., 2022). *Aspergillus* was positively correlated with soluble sugar content in tubers, highlighting its potential in carbohydrate metabolism (Davidson et al., 2011). These findings emphasize the crucial role of microbial communities in influencing potato growth and quality, and the need to manage these communities for optimal agricultural outcomes.

Although our study provides valuable insights into the impacts of legume crop rotations on soil microbial communities and plant growth, it is important to note that our findings are based on metagenomic profiling, which offers inferences about microbial functions but lacks direct validation through functional enzyme activity, transcriptomics, or soil chemical assays. Future research should aim to validate these findings through qPCR or enzymatic assays (e.g., nitrate reductase, cellulase) to confirm the activity of the identified microbial genes and their contribution to nutrient cycling. Moreover, incorporating soil chemical analyses will allow for a more comprehensive understanding of how microbial community shifts influence soil nutrient dynamics and crop productivity.

## 5 Conclusion

This study demonstrates that short-term legume rotation can improve soil microbial community structure, functional gene potential, and potato yield and quality. Among the treatments, the potato-faba bean rotation (T2) showed the strongest effects, increasing the proportion of large tubers by 85.8% and overall yield by 28.4%. Legume rotation enriched beneficial bacterial taxa, including Actinobacteria and Proteobacteria, and increased the relative abundance of genes associated with carbon, nitrogen, and sulfur metabolism, indicating enhanced potential for nutrient cycling. Potato quality was also improved, with higher starch and vitamin C contents and reduced sugar levels. Co-occurrence network analysis suggested more complex microbial interactions under legume rotations; however, these relationships are correlative and should be interpreted cautiously. While yield improvements likely result from multiple factors, including nitrogen input from legumes, associated microbial shifts may contribute complementarily. Although our findings are based on a semi-arid loamy soil, the principles of legume-facilitated nutrient enrichment and microbial regulation may be relevant to other soils and climates. Overall, legume rotation provides a practical approach for enhancing soil health and crop productivity through ecological management of the rhizosphere.

## Data Availability

The sequence data for this article is available under the accession number PRJNA1390613 at the following link: https://www.ncbi.nlm.nih.gov/bioproject/PRJNA1390613.

## Footnotes

1
https://github.com/OpenGene/fastp

2
http://www.bioinformatics.org/cd-hit/

3
https://www.ncbi.nlm.nih.gov/nr

4
https://www.genome.jp/kegg/

5
http://www.diamondsearch.org/index.php

6
www.majorbio.com

7
https://cytoscape.org/
