# Supplementary material for: Metagenomic insights into short-term legume rotation: modulating potato rhizosphere microbiota to enhance tuber yield and quality
Source: Front Microbiol. 2026 Jan 14;16:1680056. doi: 10.3389/fmicb.2025.1680056 (PMC12847424; doi:10.3389/fmicb.2025.1680056)
Supplement: Supplementary file 1 [file Data_Sheet_1.docx]

**
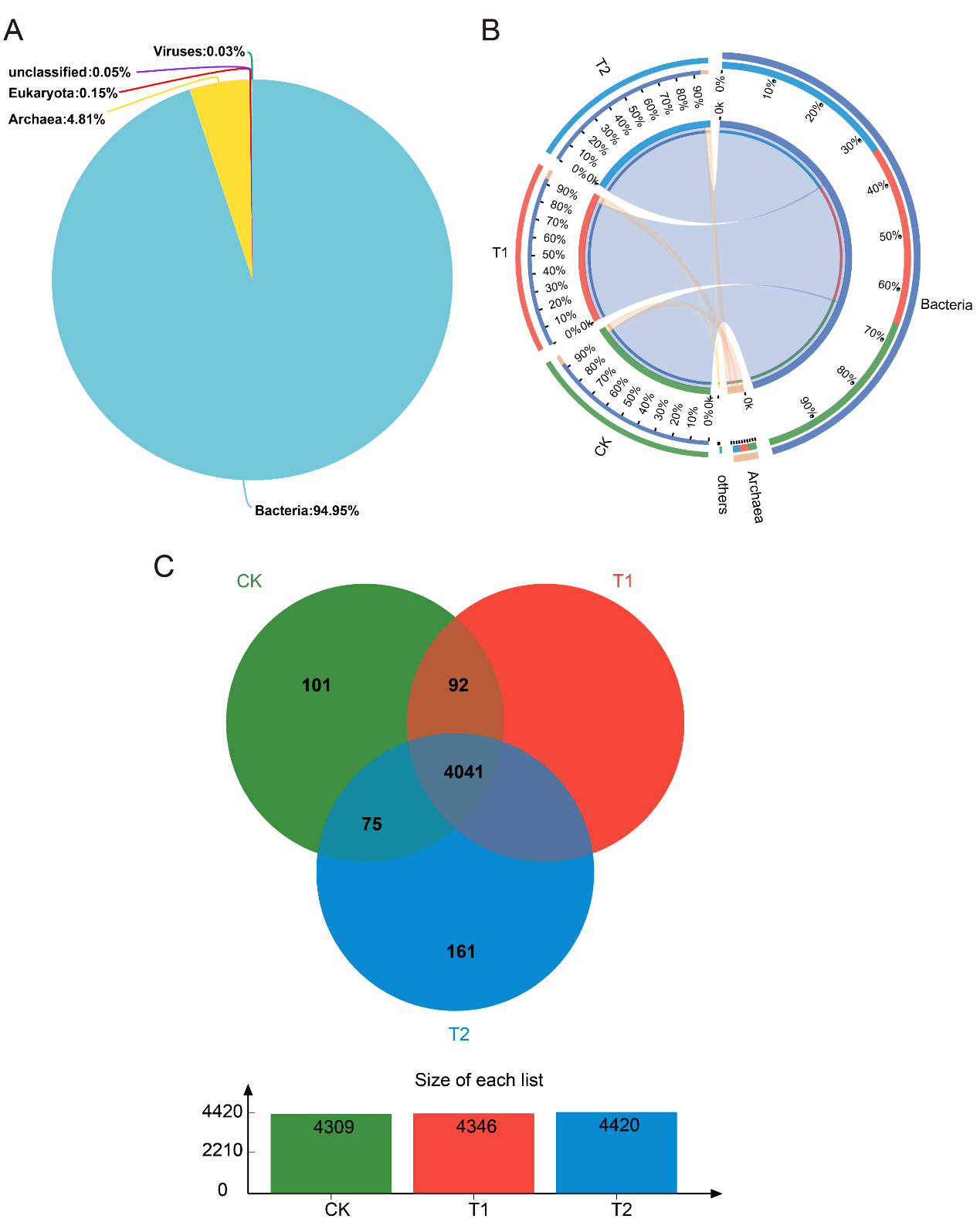
Figure S1 Composition of the soil microbial community**

Note: A represents the composition of microorganisms in the NR database; B represents the proportion of each processing; C represents the Venn diagram.

**
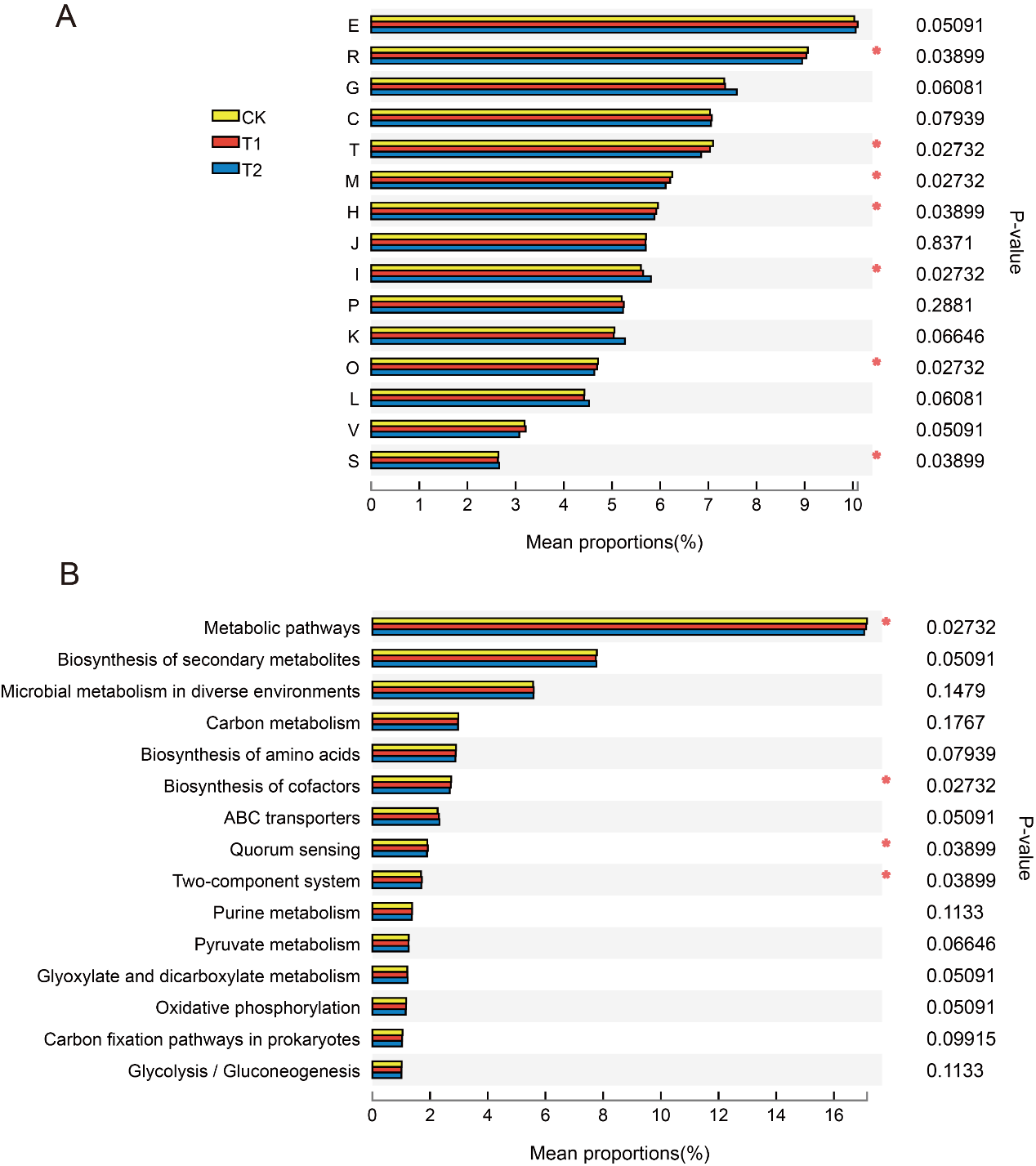
Figure S2 Functional difference test of COG (A) and KEGG (B) among soil samples**

**Table S1 Soil properties in the test area**

| Soil depth（cm） | pH | EC（μS/cm） | SOM（g/kg） | TN（g/kg） | TP（g/kg） | TK（g/kg） | Fe（mg/kg） | Ca（mg/kg） | Mg（mg/kg） |
| --- | --- | --- | --- | --- | --- | --- | --- | --- | --- |
| 0-20 | 7.05 | 329 | 12.3 | 0.96 | 0.44 | 13.56 | 6.56 | 18.33 | 0.68 |

Note: Electrical conductivity (EC), organic matter (SOM), total nitrogen (TN), total phosphorus (TP), total potassium (TK), iron (Fe), calcium (Ca), and magnesium (Mg).

**Table S2 Statistical table of sequencing results of different soil treatments**

| Sample | Raw reads | Clean reads | Percent in raw reads (%) | Contigs | Contigs bases (bp) | N50(bp) | N90(bp) | Max(bp) |
| --- | --- | --- | --- | --- | --- | --- | --- | --- |
| CK-1 | 72724888 | 71325142 | 98.07529 | 717717 | 365116633 | 503 | 333 | 16397 |
| CK-2 | 72185450 | 70881558 | 98.19369 | 697906 | 354885051 | 502 | 333 | 65178 |
| CK-3 | 74119622 | 72767960 | 98.17638 | 715158 | 366452147 | 507 | 334 | 24931 |
| T1-1 | 74003752 | 72731744 | 98.28116 | 745824 | 378396185 | 502 | 333 | 59623 |
| T1-2 | 77467262 | 76093978 | 98.22727 | 831097 | 422281956 | 500 | 333 | 18737 |
| T1-3 | 68157346 | 66845608 | 98.07543 | 663218 | 333114112 | 495 | 332 | 17062 |
| T2-1 | 70876614 | 69526416 | 98.095 | 701274 | 347644561 | 491 | 332 | 109644 |
| T2-2 | 73864684 | 72520716 | 98.1805 | 771872 | 383011043 | 489 | 332 | 14787 |
| T2-3 | 84406918 | 82776800 | 98.06874 | 929997 | 457872432 | 483 | 331 | 40982 |

Note: CK, continuous potato cropping; T1, potato-pea rotation; T2, potato-broad bean rotation.
